# How can patient partnership help to improve equality as COVID‐19 moves from pandemic to endemic?

**DOI:** 10.1002/lim2.74

**Published:** 2022-12-29

**Authors:** Natasha Treagust

**Affiliations:** ^1^ School of Clinical Medicine University of Cambridge Cambridge UK

**Keywords:** diversity equality inclusion, patient involvement, patient‐focused care

## Abstract

One of the stated aims of the National Health Service (NHS) within its constitution is the promotion of equality. However, there is evidence inequality has increased over the last decade and the COVID‐19 pandemic has exacerbated this. The current pressures on healthcare mean that there is a case for a shift in approach as we transition to living with endemic COVID‐19. This article sets out how putting patient partnership front and centre at an individual, systems and national level has the potential to improve equality and assist the NHS in achieving its founding principles.

The National Health Service (NHS) aims to provide a ‘comprehensive service, available to all’ and has a ‘wider social duty to promote equality through the services it provides’.^1^ However, there is evidence from the last decade that inequality is increasing. For example, Public Health England data show from 2010–2012 to 2016–2018 women in the least deprived six deciles gained 0.5 years of life expectancy, while those in the most deprived decile lost 0.3 years.^2^ The COVID‐19 pandemic has further exacerbated inequalities in several ways. Higher mortality rates were experienced by individuals from more deprived areas.^3^ Further to this, impacts of lockdown restrictions on the wider determinants of health, for example education, are being felt disproportionately by those who were already the most disadvantaged in our society.^4^

Healthcare's transition from a pandemic footing is more than a theoretical line in the sand. Tackling COVID‐19 loaded additional demands on a system already under pressure. There is now a wide gap between public expectations and what we can provide.^5^ Consequently, more than ever, there is a case for a shift in approach. Here, I will set out how putting patient partnership front and centre, levelling the power balance between patients, doctors and executives, has the potential to improve equality and assist the NHS in achieving its founding principles.

Medical school teaches us the biomedical underpinnings of a plethora of diseases. In theory, forming a differential diagnosis based on a patient's history and examination enables doctors to offer treatments and relieve suffering. However, an individual's health is a fluid point on a continuum, influenced by much more than the list of symptoms and signs we elicit. Each person is a unique intersection of wider determinants that range from housing to employment.^6^ These will impact their vulnerability to illness, access to healthcare and the efficacy of treatments. Failing to account for this in interactions with patients has the potential to exacerbate inequalities. The minority who comfortably fit our disease models and have the resources needed to access care will likely have good outcomes. However, many will not fit these criteria. At best some will derive less benefit from treatment. However, for others, an unconsidered approach may precipitate indirect harm. For example, being unable to access treatment due to a lack of transport may increase feelings of exclusion. The psychological effects of this have the potential for wider negative implications for their life. Hope Citadel, a group of primary care practices in Greater Manchester, demonstrates how active partnership with patients can mitigate this.^7^ They aim to offer care that considers the whole person. Where possible, they treat individuals in the community and have developed pathways so practitioners can go beyond firefighting patients’ immediate symptoms and address underlying causes. Approaches such as this have the potential to improve equality by directly tackling the disproportional vulnerability of some groups to ill health.

At a systems level, patient partnership is equally vital. People are living longer and have increased levels of multimorbidity.^2^ Healthcare needs a long‐term plan with clear goals, the path to which is mapped through resourced, delineated steps. It verges on arrogance to assume a single group has sufficient understanding of the goals, aims and desires of all who comprise our society to plan equitable care for a post‐pandemic world. The co‐production of services is one way this diversity can be accounted for. Voices for improvement^8^ is an example of where this is being implemented. ‘Lived experience partners’ are given training which enables them to coach healthcare providers. The partnership of patients and doctors that this creates enhances doctors’ awareness of the variety of patient perspectives, expanding the breadth of their considerations when meeting future patients. Projects such as these have the potential to improve equality. They increase the chance due respect is given to both the wider circumstances of patients and the outcomes that are important to them. Through this more people should be able to derive benefits from available healthcare.

At the national level, the current restructuring of the NHS presents an opportunity to further integrate patient partnership. Services are now organised under integrated care systems (ICSs). As part of this, health inequalities are being addressed by a scheme known as Core20PLUS5. This funds ICSs to recruit ‘connectors’ from disadvantaged communities who can voice the challenges people face day to day.^9^ These partnerships should allow patients to influence the future direction of the NHS and enhance the design of healthcare, so it meets the needs of local people. The tailored services developed as a result hope to improve equality by reducing differences in outcomes between communities.

These examples have shown how a partnership with patients can improve health equality. However, health is one element of inequality, and altering the power balance between the system and the people it serves could have greater benefits. Inequalities cluster and have reciprocal effects on each other.^10^ Consequently, partnering with patients to develop services may have a positive effect on many more aspects of their lives than those immediately associated with health. In addition to this, increasing the prominence of patient partnership has the potential for psychological benefits. The feeling systems are not designed to work for you, or your voice does not matter and resonates with those who experience many types of inequality: racial, age, economic and health. Showing we are doing more than paying lip service to individuals through acting in partnership with our patients and shaping systems in the light of their feedback would be an important step towards countering this. One example which supports this comes from work by the King's Fund looking at the benefits of the co‐production of services for people with disabilities.^11^ Meaningful coproduction was found to increase participants’ self‐esteem, confidence and sense of agency.

In conclusion, patient partnership can be a powerful means of improving equality as we move from pandemic to endemic COVID‐19. This would be achieved not only as a product of the work of partnerships. The very act of committing to patient partnership would be a step towards greater equality. It would demonstrate how the values and experiences of all in our society are equally important to directing where our NHS goes next.

## CONFLICT OF INTEREST

The author declares no conflict of interest.

## FUNDING INFORMATION

This essay was the winner of the Royal Society of Medicine, ‘Medicine and Society Section: Doubleday Student Prize 2022’. The cost of publishing the manuscript open access was part of the prize.

## Data Availability

No data were collected and analysed specifically to produce this manuscript. All sources of information are linked in the bibliography.
